# Redefining care for IDH-mutant low-grade gliomas: Clinical implications of the INDIGO trial and updated guidelines

**DOI:** 10.1016/j.clinme.2026.100644

**Published:** 2026-09-17

**Authors:** Kristin S. Bartman, Katherine B. Peters

**Affiliations:** Preston Robert Tisch Brain Tumor Center, Department of Neurosurgery, Duke University School of Medicine, Durham, NC, United States

**Keywords:** Isocitrate dehydrogenase (IDH), IDH mutant (mIDH) gliomas, Astrocytoma, Oligodendroglioma, INDIGO trial, Vorasidenib, Progression free survival (PFS), Time to next intervention (TTNI)

## Abstract

Pathogenic variants in the genes encoding isocitrate dehydrogenase (IDH1 and IDH2) are the defining driver mutations in diffuse low-grade gliomas. When IDH is mutated, the production of the oncometabolite 2-hydroxyglutarate triggers downstream metabolic changes that promote gliomagenesis. The identification of the isocitrate dehydrogenase mutation (mIDH) in diffuse gliomas has not only redefined prognosis but also treatment for patients with these tumours. With the identification of the IDH mutation, targeted therapies have emerged as a promising therapeutic strategy in gliomas. The INDIGO trial compared the IDH inhibitor vorasidenib to a placebo. Progression-free survival (PFS) and time to next intervention were extended in patients who received vorasidenib vs. placebo. The side-effect profile was tolerable compared to that of traditional cytotoxic chemotherapies. In August 2024, vorasidenib received FDA approval for the treatment of grade 2 mIDH gliomas and is included in the National Comprehensive Cancer Network Guidelines for central nervous system tumours.

## Highlights

• Mutations in the gene isocitrate dehydrogenase 1 and 2 can occur in diffuse gliomas and are enriched in low-grade gliomas.
• Astrocytomas and oligodendrogliomas are classified as IDH mutant gliomas.
• The INDIGO trial was a phase III, randomised, double-blinded study evaluating the effect of the oral IDH inhibitor, vorasidenib, in patients with grade 2 mIDH gliomas.
• Progression-free survival and time to next intervention were significantly longer in the vorasidenib treatment group.
• Treatment with vorasidenib did not affect quality of life or have significant toxicity.

## Graphical Abstract

**Figure:**
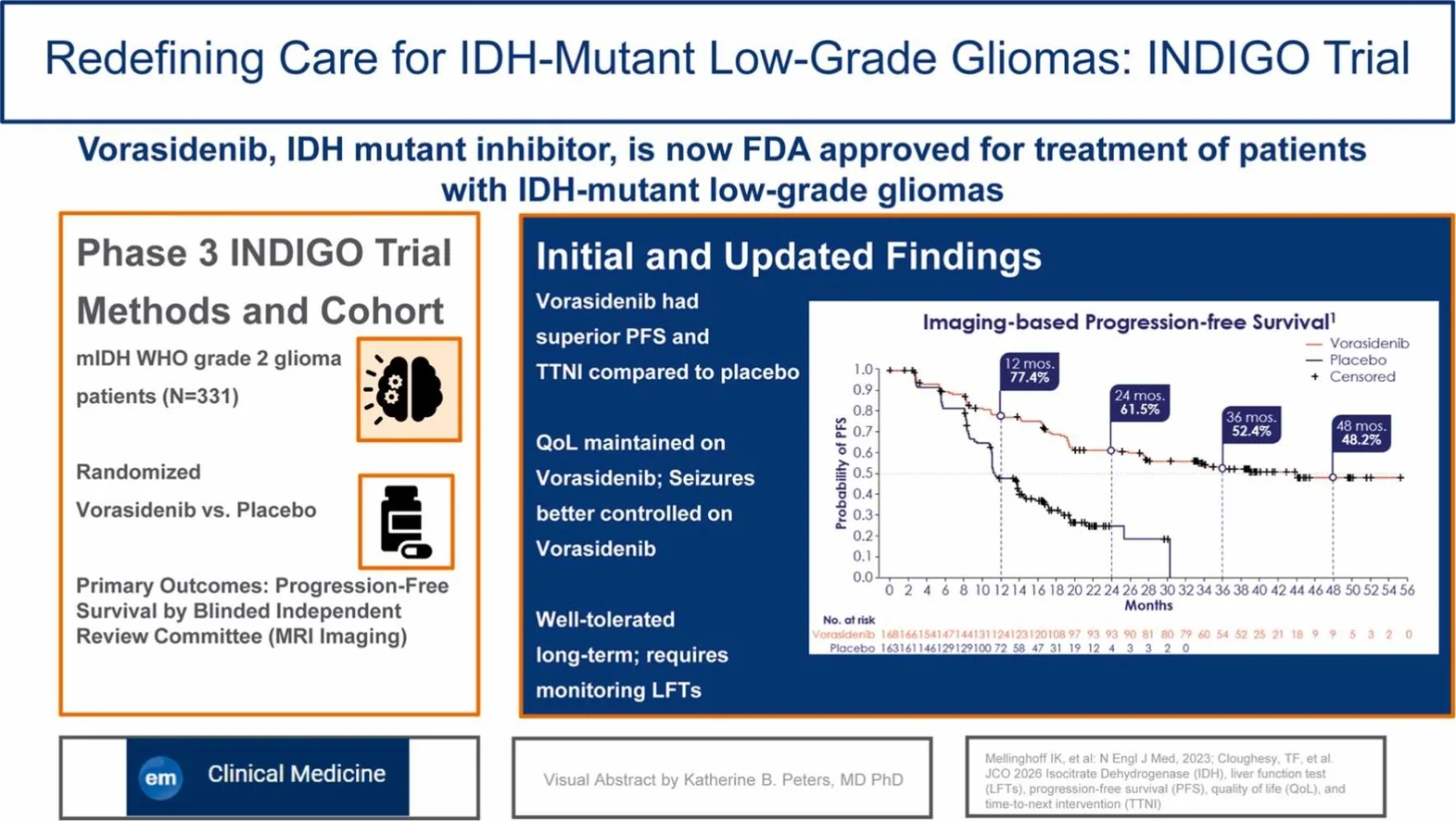

## Background

Pathogenic variants in the genes encoding isocitrate dehydrogenase (IDH1 and IDH2) are a common driver mutation in diffuse low-grade glioma.^1^ The identification of the isocitrate dehydrogenase mutation (mIDH) prompted the reclassification of diffuse gliomas. In 2016, the World Health Organization (WHO) incorporated IDH mutations into the classification of gliomas.^2^ This designation was further refined in the 2021 WHO classification of mIDH astrocytomas and mIDH oligodendrogliomas from IDH wild-type glioblastoma.^3^ Adult diffuse type gliomas were reclassified into mIDH astrocytoma (WHO grades 2, 3, 4), mIDH 1p/19q co-deleted oligodendroglioma (WHO grades 2, 3), and IDH wild type glioblastoma (WHO grade 4). CNS tumour grade is no longer entirely histopathologic; the presence of molecular *CDKN2A* and/or *CDKN2B* homozygous deletion results in a CNS grade 4 designation for these tumours, even in the absence of microvascular proliferation or necrosis, signalling a significant shift in their grading and treatment.^4^

Historically, patients with low-grade diffuse gliomas were treated with standard-of-care therapy involving upfront maximally safe resection, followed by radiation and chemotherapy with consideration of regular MRI surveillance if patients were deemed to be low risk (young patients less than 45 years of age, who underwent a gross total resection, and did not have significant neurological deficits).^5^ Unfortunately, these treatment strategies are known to carry significant long-term toxicity and neurocognitive deficits that affect quality of life.^6^, ^7^, ^8^ The identification of the IDH mutation in these tumours enabled physicians to stratify treatment options and prognosis in gliomas.

Pathogenic variants in the IDH 1/2 genes produce the oncometabolite 2-hydroxyglutarate, which plays a key role in gliomagenesis in these tumours.^9^ Nearly 90% of all pathogenic variants in IDH1 gene are a R132H substitution (arginine replaced by histidine at the codon 132). Other less frequent pathogenic variants include R132C, R132G, R132S, and R132L.^10^ Pathogenic variants of IDH2 gene are less commonly found in gliomas (roughly 6%) and included common alterations at R172K and R172M.^11^ Given IDH specific pathogenic variants play a role in gliomagenesis, targeted therapies have emerged as a promising therapeutic strategy.

Their successful application in other malignancies has informed the clinical development of IDH inhibitors in glioma. Ivosidenib, an orally administered, selective inhibitor of mutant IDH1, was approved by the FDA in 2018 for patients with relapsed or refractory mIDH1 acute myeloid leukaemia.^12^ Its activity in mIDH1 gliomas was subsequently evaluated in two clinical studies. In a phase I dose-escalation trial, 66 patients with recurrent mutant IDH1 gliomas received ivosidenib at escalating doses. Ivosidenib demonstrated a favourable safety profile, with no dose-limiting toxicities, and 500 mg once daily was established as the maximum tolerated and recommended dose.^13^ A subsequent study incorporated a perioperative design to assess intratumoral drug activity and pharmacodynamic effects directly.^14^ At the 500-mg once-daily dose, ivosidenib produced a measurable reduction in intratumoral 2-hydroxyglutarate, confirming intratumoural target engagement. However, comparative analyses demonstrated that ivosidenib did not achieve superior tumour penetration relative to vorasidenib, a next-generation, orally available dual mIDH1/2 inhibitor. These findings contributed to selecting vorasidenib for further clinical development, culminating in the phase III INDIGO trial (NCT04164901).^15^

Vorasidenib is an oral, brain-penetrant inhibitor of mutant IDH1/2 designed to target this molecular driver.^16^ Phase 1 study of vorasidenib in recurrent IDH1 or IDH2 mutant glioma patients established a 50-mg orally once-daily dose with a favourable safety profile.^17^ Ivosidenib and vorasidenib were then compared head-to-head in the forementioned perioperative study by Mellinghoff and colleagues.^14^ Vorasidenib provided superior blood-brain barrier penetration and significantly increased intratumoural penetration, hence vorasidenib was chosen for further development. The pivotal phase III INDIGO trial compared vorasidenib to placebo.^15^ In August 2024, vorasidenib received FDA approval in the U.S. for the treatment of grade 2 mIDH1/2 gliomas and is also approved in Japan, Australia, and some European countries.

## Trial objectives and design

The INDIGO trial aimed to determine whether the oral brain-penetrant IDH inhibitor, vorasidenib, affected progression-free survival as assessed by MRI in patients with measurable grade 2 mIDH gliomas.^15^ This clinical trial was a phase III, international, randomised, double-blind study. The design was 1:1 placebo-controlled. Patients aged 12 and older with measurable non-enhancing disease (>1 cm × 1 cm), histology-proven, IDH-mutant 1 or IDH-mutant 2 WHO grade 2 gliomas were included. All patients had been treated with surgery only and had received no prior radiation or chemotherapy. Radiographic evidence of measurable non-enhancing disease and clinical stability, in particular attention to seizures that had to be under control, at enrolment, were required. Secondary and exploratory endpoints evaluated health-related quality of life, neurocognitive function, seizure control, and tumour response. The Functional Assessment of Cancer Therapy-Brain (FACT-Br) questionnaire evaluated health-related quality of life. Neurocognitive testing assessed cognitive function. Reported seizure frequency was monitored and obtained per patient diary. MRI volumetrics were used to assess tumour growth.

## Results

Median progression-free survival (PFS) with vorasidenib was significantly longer than with placebo: 27.7 months vs. 11.1 months, with a hazard ratio of 0.39. Time to the next intervention was also extended, and the median time to the next intervention has not been reached in the treatment arm, with a hazard ratio of 0.26, compared with 17.8 months in the placebo group. Secondary and exploratory endpoints, including health-related quality of life, neurocognitive function, seizure control, and tumour growth, were evaluated. Baseline Fact-Br was high in both groups, and there was no meaningful deterioration in Fact-Br scores in the treatment group during the study period. Cognitive outcomes in both treatment groups were comparable during the study period. This point is critical to note given that the need for subsequent therapies that can impact negatively on cognition, such as radiation therapy, are delayed significantly when one looks at time-to-next-intervention in this study. Seizure frequency was reduced in the treatment group compared to placebo: 18.2 seizures per person per year vs 51.2 seizures per person per year. In the placebo group, tumours continued to grow throughout the study period, with an average growth rate of 14.4%, whereas tumours treated with vorasidenib had an estimated growth rate of −1.3%.

At the 2026 American Society of Clinical Oncology Annual Meeting, investigators presented an extended follow-up analysis of the Phase 3 INDIGO trial evaluating vorasidenib in patients with grade 2 IDH-mutant glioma who did not yet require radiation or chemotherapy.^18^ Notably, the median follow-up time was 41.6 months for this analysis. Compared with the original 2023 results from the NEJM publication, the longer follow-up (median follow-up time of 41.6 months) showed that the median PFS for patients on vorasidenib was 44.1 months (95% CI 27.7, NR). Moreover, the median time to the next intervention had not yet been reached, with only 23.8% of patients requiring subsequent treatment with other modalities such as radiation therapy or chemotherapy. One key exploratory endpoint was seizure control and included data from patients who had at least one seizure during the clinical trial showed a 72% reduction in on-treatment seizure rate for patients that received vorasidenib. Seizure control appeared particularly pronounced in patients with oligodendroglioma. Importantly, there were no new long-term safety signals associated with vorasidenib; the most common adverse events were elevations in liver enzymes (ALT and AST), which were typically grade 1.

## Discussion

The INDIGO trial has shifted the treatment strategies for mIDH gliomas. The results of the INDIGO trial support the use of vorasidenib to delay tumour growth and postpone radiation and chemotherapy. Median progression-free survival is significantly longer with vorasidenib than with placebo, and time to next intervention is still ongoing in the treatment group. Exploratory endpoints showed that vorasidenib did not significantly affect health-related quality of life or neurocognitive function. Seizure frequency decreased in the vorasidenib treatment group, and volumetric analysis suggested a potential reduction in tumour volume with vorasidenib. In addition, for those with grade 2 mIDH tumours previously considered for observation, there are now early therapeutic intervention options for these patients.

Delaying the need for cytotoxic chemotherapy and radiation therapy is statistically meaningful in this population, as many are young adults, and traditional therapies have known toxicities and effects on neurocognition. While there are limitations, this pivotal Phase III trial has opened the door to exploring the use of IDH inhibitors in mIDH gliomas, and vorasidenib is now incorporated in the NCCN guidelines for grade 2 and 3 mIDH tumours. Further research is needed to assess overall survival, the long-term effects, and use in broader mIDH glioma populations (Table 1).

**Table 1:** Side-by-side comparison of the original 2023 INDIGO presentation/publication versus the 2026 ASCO extended follow-up.

Outcome	2023 INDIGO (ASCO/NEJM)^15^	2026 ASCO Extended Follow-up^18^	What Changed?
**Median follow-up**	14.2 months	41.6 months	Nearly three years of additional observation.
**Median progression-free survival (PFS)**	27.7 months	44.1 months	The estimated median PFS increased by more than 16 months, demonstrating durable disease control with longer follow-up.
**Hazard ratio for progression/death**	0.39 (61% risk reduction vs placebo)	Randomised comparison less informative after placebo crossover	Because almost all placebo patients crossed over to vorasidenib after unblinding, long-term HR comparisons are no longer the primary focus.
**Time to next intervention (TTNI)**	Not reached; HR 0.26	Median still not reached; only 23.8% required another treatment	Confirms that many patients continue to avoid radiation or chemotherapy for years.
**Objective response rate (ORR)**	Approximately 11%	20.8%	Responses continued to accumulate over time, suggesting gradual tumour shrinkage in additional patients.
**Seizure outcomes**	Not a major efficacy endpoint	72% reduction in on-treatment seizure rate	One of the most clinically meaningful new findings, especially for symptomatic patients.
**Safety**	Mainly reversible liver enzyme elevations; few discontinuations	No new long-term safety signals	Long-term therapy remained generally well tolerated.

## CRediT authorship contribution statement

**Katherine B. Peters:** Writing – review & editing, Writing – original draft, Conceptualization. **Kristin S. Bartman:** Writing – review & editing, Writing – original draft, Conceptualization**.**

## Funding

This research did not receive any specific grant from funding agencies in the public, commercial, or not-for-profit sectors.

## Declaration of competing interest

The authors declare the following financial interests/personal relationships which may be considered as potential competing interests: Katherine B. Peters reports a relationship with Servier Pharmaceuticals LLC that includes: consulting or advisory and funding grants. Katherine B. Peters reports a relationship with Nuvation Bio, Inc that includes: consulting or advisory and funding grants. Katherine B. Peters reports a relationship with Rigel Pharmaceuticals Inc that includes: consulting or advisory. If there are other authors, they declare that they have no known competing financial interests or personal relationships that could have appeared to influence the work reported in this paper.
